# Hepatic lipidosis in nine African white-bellied pangolins (*Phataginus tricuspis*) from a North American zoologic institution

**DOI:** 10.3389/fvets.2025.1562904

**Published:** 2025-06-03

**Authors:** Molly Horgan, Jennifer Landolfi, Nicole I. Stacy, Jennifer Watts, Copper Aitken-Palmer

**Affiliations:** ^1^Department of Comparative, Diagnostic, and Population Medicine, College of Veterinary Medicine, University of Florida, Gainesville, FL, United States; ^2^Zoological Pathology Program, University of Illinois, Brookfield, IL, United States; ^3^Department of Large Animal Clinical Sciences, College of Veterinary Medicine, University of Florida, Gainesville, FL, United States; ^4^Brookfield Zoo Chicago, Brookfield, IL, United States

**Keywords:** pangolin, *Phataginus tricuspis*, hepatic lipidosis, liver, anorexia, obesity

## Abstract

**Introduction:**

The African white-bellied pangolin (*Phataginus tricuspis*) is an endangered species with a small population under managed care in the United States. Over the course of 4 years, nine pangolins at a single North American zoological institution died or were euthanized with necropsy findings consistent with hepatic lipidosis, representing 9 of 14 mortalities during this time period.

**Methods:**

This report describes hepatic lipidosis in these pangolins (clinical presentation, diagnostic imaging, clinicopathologic and postmortem findings) and summarizes clinically relevant predictors of disease.

**Results:**

The time from the onset of illness to death ranged from 2 to 75 days (median 23 days). Obesity was noted prior to clinical presentation for illness in 4/9 animals. All pangolins had anorexia and lethargy; other common clinical signs included constipation (6/9), vomiting or regurgitation (6/9), and/or dyspnea (5/9). Increased alanine aminotransferase (ALT) and aspartate aminotransferase (AST) were observed in 7/9 animals, hyperbilirubinemia in 5/9, and increased bile acids in 5/9. Serum selenium was low in 3/9 animals, but vitamin E concentrations were normal. Hypertriglyceridemia was uncommon during illness (1/9). Evidence of renal dysfunction was also common, and included glucosuria (6/6), proteinuria (7/7), and azotemia (6/9). Ultrasound was the most reliable imaging technique at identifying hepatic lipidosis as evidenced by hepatomegaly and increased echogenicity. Despite variable treatment including assisted feeding, hepatoprotectants, fluid therapy, gastroprotectants, antibiotics, and/or antiemetics, all cases were ultimately fatal. Each of the animals had comorbidities or an inciting reason for anorexia that led to negative energy balance. In 5/9, resultant hepatic lipidosis was severe and deemed the primary cause of death or euthanasia.

**Discussion:**

This case series suggests that white-bellied pangolins are prone to developing hepatic lipidosis following a period of anorexia secondary to other underlying disease processes. Elevated AST, ALT, bilirubin, and bile acids in an anorectic and lethargic pangolin should provide a high index of suspicion for hepatic lipidosis. Further study is needed to evaluate pangolins for potential unique polyunsaturated fatty acid or other species-specific dietary requirements.

## Introduction

Pangolins (family Manidae, order Pholidota) include eight distinct species, all of which are classified by the International Union for the Conservation of Nature (IUCN) as vulnerable, endangered, or critically endangered, in large part due to trafficking for the traditional medicine market (1, 2). The African white-bellied pangolin (*Phataginus tricuspis*) is an endangered species native to central and West Africa. In 2016, a group of white-bellied pangolins was exported from West Africa to zoological institutions in the United States with the goals of advancing research and establishing a stable captive breeding program for conservation purposes (3).

*Ex-situ* populations of pangolins also face challenges, with historically poor survival rates in zoos (4). They are myrmecophagous, making their diets difficult to replicate (4, 5). With some success, recent studies have investigated methods of feeding animals under managed care that more closely replicate their diet in the wild with improved palatability and digestibility (5–7). Given the endangered status of these animals, documenting causes of morbidity and mortality in managed care is crucial. Published information on diseases of African pangolins is lacking. Reported causes for morbidity and mortality in Asian pangolin species under managed care include pneumonia, trauma, and gastrointestinal and renal disease (8–10). Hepatobiliary disease has also been reported, with one retrospective study of necropsy findings in rescued Formosan pangolins reporting a 43% (6/14) incidence of hepatic lesions, 67% (4/6) of which included some degree of hepatic lipidosis (10).

Hepatic lipidosis has been a significant contributor to mortality of white-bellied pangolins at our institution, and a threat to the goal of establishing a stable *ex-situ* population. This retrospective report details the clinical signs, treatments, as well as clinicopathologic, diagnostic imaging, and postmortem findings in nine white-bellied pangolins from a single zoological facility in the United States that died or were euthanized with hepatic lipidosis.

## Materials and methods

The medical records of nine white-bellied pangolins (*Phataginus tricuspis*) from a single zoological institution with the postmortem diagnosis of hepatic lipidosis from 2016 to 2019 were examined retrospectively. Initiation of illness was assigned as the date of the first exam performed by a veterinarian due to signs of illness to which the animal eventually succumbed. Recorded information included signalment, the source of the animal, length of current residency, husbandry details, diet, date of initial illness, clinical signs, clinicopathologic data, imaging results, treatments, clinical outcome, gross necropsy findings, and histologic lesions. Hematology and serum biochemistry reference intervals (RI) for white-bellied pangolins from the Zoological Information Management System (ZIMS) (11) were used, or when these were not available, extrapolated from domestic animals. Gross and histologic necropsy findings were extracted from reports generated by American College of Veterinary Pathologist board certified professionals. Hepatic lipidosis was diagnosed histologically when hepatocellular lipid accumulation resulted in distortion of normal architecture. Features included large lipid vacuoles with displacement and/or compression of the nuclear profile and indistinct cellular margins with apparent coalescence of lipid vacuoles in adjacent hepatocytes. The cause of death or reason for euthanasia was determined by the pathologist performing the necropsy as the most significant disease process leading to demise.

Additionally, select banked serum samples were analyzed for triglycerides, bile acids, and protein electrophoresis (University of Miami Comparative Pathology Laboratory, Miami, FL), selenium (Michigan State University Diagnostic Laboratory, Lansing, MI), and vitamin E (Heartland Assays, Ames, IA) to gain a better understanding of how these analytes changed during the course of illness. If available, this was done on samples from a period of health (baseline), at the start of illness (early illness), and as close as possible to death (late illness).

## Results

### Husbandry

All pangolins were housed and cared for in a similar manner with professional zoological trained staff. Males and females were housed separately except when doors of adjacent enclosures were opened for breeding encounters. Females were housed with offspring until weaning. All pangolins had access to climbing structures and at least one nest box. They were maintained on a reverse light cycle with timed lights controlled for 12 h of daylight. The enclosures were climate controlled with temperature and humidity monitors to mimic a tropical climate.

### Nutrition

During the study period, animals were individually fed twice daily, with amounts based on body weight, caloric intake, and behavior. The majority (>50%) of the diet by dry weight was composed of dried insects, including bottle and soldier fly larvae, mealworms, silkworms, crickets, and black ants. Other additives included flax seed, brewer’s yeast, powdered chitin, dried powdered spirulina, formic acid, soil and calcium carbonate. Nutrient composition of the diet was based off current knowledge for carnivores and insectivores, including other species of pangolin (5–7, 12). Water was added to the diet for a water weight ratio of 160%. Adjustments to the diet were made frequently from 2016 to 2020; including changes to fat-soluble vitamins supplementation, adding a mineral supplement due to low serum zinc and copper, adding B_12_ and taurine, and changing type of soil fed based on high serum iron concentrations.

### Case summaries

From 2016 to 2019, there were 14 *P. tricuspis* mortalities at the institution including two still births. Of these, nine animals (three zoo born, six wild born) had a diagnosis of hepatic lipidosis at necropsy and fit the case definition for inclusion in this report. Summaries of signalment, clinical signs, clinicopathologic findings, and histopathology are given in Tables 1–4.

**Table 1:** Signalment, body condition, weight loss history, and information on initial clinical signs and inciting events for anorexia in nine white-bellied pangolins (*Phataginus tricuspis*) diagnosed with hepatic lipidosis at necropsy.

Case number	Sex	Age at time of death (months)	History of obesity	Length of time from onset of illness to death (days)	Percentage weight loss from initial illness to death	Body condition at death	Initial clinical sign	Inciting event or comorbidity leading to anorexia
1	F	Adult	No	19	13.5%	Good	Inappetence, lethargy	Pregnancy, lactation
2	M	9	No	12	0.9%	Good	Regurgitation	Megaesophagus, pneumonia
3	F	Adult	Yes	23	14.5%	Obese	Inappetence, hematuria	Cystitis
4	F	Adult	No	37	12.2%	Good	Inappetence, lethargy, decreased fecal production	Intestinal parasitism (acanthocephalans), pancreatitis
5	M	Adult	Yes	11	10.4%	Good	Inappetence, decreased fecal production	Pyloric stenosis, aspiration pneumonia
6	M	7	No	31	10.8%	Good	Inappetence	Chronic stress^*^, megaesophagus, pancreatitis
7	M	46	No	2	-	Good	Dyspnea	Pneumonia
8	F	Adult	Yes	75	20.7%	Fair	Inappetence	Pregnancy, lactation
9	F	Adult	Yes	53	6%	Good	Inappetence, vomiting	Mammary gland abscess, Clostridial enteritis

^*^Construction in a nearby enclosure provided presumed source of stress that manifested as abnormal behavior as compared to others.

**Table 2:** Data on hepatobiliary leakage enzymes, hepatic functional parameters, and triglycerides from nine white-bellied pangolins (*Phataginus tricuspis*) diagnosed with hepatic lipidosis at necropsy.

Case number	Timepoint	ALT (28–205 IU/L)	AST (29–136 IU/L)	GGT (0–18 IU/L)	ALP (30–184 IU/L)	Total bilirubin (0.1–1.0 mg/dL)	Bile acids^1^ (<30 μmol/L)	Triglycerides^1^ (21–120 mg/dL)
1	Baseline	67	39	5	50	0.3	**37**	4.2
	Early illness	**214**	**811**	6	62	0.7	n/a	n/a
	Late illness	193	**351**	7	46	**5.4**	**58.8**	n/a
2	Baseline	28	27	3	120	0.5	26.8	39
	Early illness	85	61	9	66	0.7	26.7	17
	Late illness	133	n/a	n/a	98	0.4	**30.7**	12
3	Baseline	61	39	5	42	0.3	8	27
	Early illness	**387**	**437**	18	105	0.6	**37.3**	33
	Late illness	**308**	**1,083**	**39**	**249**	**8.4**	**309.8**	106
4	Baseline	130	59	4	90	0.1	**53.8**	**229**
	Early illness	**337**	**294**	5	83	0.6	**53.5**	40
	Late illness	**236**	**179**	6	108	**1.4**	**34.2**	27
5	Baseline	79	77	3	76	0.1	**33.9**	**174**
	Early illness	115	83	5	69	0.7	7	13
	Late illness	**235**	**249**	4	88	0.7	17.3	44
6	Baseline	49	36	3	84	0.3	12.9	23
	Early illness	**986**	**3,756**	10	122	**1.5**	25.9	20
	Late illness	139	**152**	12	89	0.6	25.4	52
7	Baseline	56	51	4	38	0.3	15.3	14
	Early illness	n/a	n/a	n/a	n/a	n/a	n/a	n/a
	Late illness	66	56	8	62	n/a	23.1	10
8	Baseline	62	53	3	51	0.1	18.2	56
	Early illness	111	100	2	107	0.6	9.1	18
	Late illness	**527**	**5,301**	**22**	**314**	**1.3**	**30.1**	**412**
9	Baseline	73	82	1	79	0.1	27	18
	Early illness	**251**	**210**	**20**	105	0.6	15.9	10
	Late illness	106	92	14	118	<0.1	9.3	30

Reference ranges from ZIMS are given in the top row (11). Values are bolded if above the reference interval. IU/L, international units per liter; mg/dL, milligrams per deciliter; μmol/L, micromole per liter; ALT, alanine aminotransferase; AST, aspartate aminotransferase; GGT, gamma-glutamyl transferase; ALP, alkaline phosphatase; n/a, parameter not measured at given timepoint. ^1^No species-specific reference intervals were available for bile acids or triglycerides, so they were extrapolated from domestic small mammals (13).

**Table 3:** Data on blood renal markers, albumin as measured by protein electrophoresis, urine protein:creatinine ratio, and urine glucose with paired serum glucose from nine white-bellied pangolins (*Phataginus tricuspis*) diagnosed with hepatic lipidosis at necropsy.

Case number	Timepoint	Creatinine (0.1–0.7 mg/dL)	BUN (9.5–49.4 mg/dL)	Serum albumin (2.05–4.28 g/dL)	UPC (0.24–0.76)	Serum glucose (45–157 mg/dL)	Urine glucose (mg/dL)
1	Baseline	0.4	19	n/a	n/a	95	n/a
	Early illness	0.3	*5*	n/a	n/a	123	n/a
	Late illness	**0.9**	32	n/a	n/a	57	n/a
2	Baseline	0.3	23	*1.94*	n/a	92	n/a
	Early illness	0.2	**63**	3.26	n/a	79	n/a
	Late illness	0.3	30	2.67	n/a	110	n/a
3	Baseline	0.1	30	4.02	0.5	84	Negative
	Early illness	0.2	**105**	4.06	**n/a** ^**1**^	**159**	**Trace**
	Late illness	n/a	**107**	3.09	n/a	123	**100**
4	Baseline	<0.1	30	3.39	n/a^2^	83	Negative
	Early illness	0.2	11	3.22	0.5	119	Negative
	Late illness	0.5	*4*	*1.9*	**2**	126	**1,000**
5	Baseline	<0.1	38	3.38	0.3	111	Negative
	Early illness	0.7	15	3.71	0.6	92	Negative
	Late illness	**1.0**	19	4.05	**4.0**	133	**100**
6	Baseline	0.2	26	3.43	0.4	104	Negative
	Early illness	0.7	*8*	4.14	**2.9**	**190**	**1,000**
	Late illness	**0.8**	15	2.64	**1.7**	**423**	**>2,000**
7	Baseline	0.3	24	2.41	**0.8**	123	Negative
	Early illness	n/a	n/a	n/a	n/a	n/a	n/a
	Late illness	0.3	16	2.54	**1.7**	131	n/a
8	Baseline	0.1	16	3.85	0.5	101	Negative
	Early illness	**0.9**	*6*	3.73	0.5	135	Negative
	Late illness	0.6	10	3.86	**3.9**	138	**>2,000**
9	Baseline	0.2	18	3.66	0.2	100	Negative
	Early illness	0.6	*3*	2.92	0.4	81	Negative
	Late illness	0.2	*7*	*1.21*	**2.1**	**179**	**1,000**

Reference ranges from ZIMS are given in the top row (11). Values are bolded if above the reference interval, and italicized if below the reference interval. mg/dL, milligrams per deciliter; g/dL, grams per deciliter; BUN, blood urea nitrogen; UPC, urine protein:creatinine ratio; n/a, parameter not measured at given timepoint. ^1^UPC not performed, but urine protein measurement was >2,000 mg/dL. ^2^UPC not performed, but urine protein measurement was negative.

**Table 4:** Summary of histopathological findings in nine-white bellied pangolins (*Phataginus tricuspis*) diagnosed with hepatic lipidosis at necropsy.

Case number	Histologic severity of hepatic lipidosis	Other hepatic lesions	Renal lesions	Pulmonary lesions	Gastrointestinal lesions	Other significant lesions	Cause of death or reason for euthanasia
1	Marked	Bile stasis	Marked tubular lipidosis Mild acute tubular proteinosis and necrosis	-	Gastric and colonic muscularis myositis and soft tissue mineralization. Mild epithelial retention in esophagus and stomach	-	Hepatic lipidosis
2	Mild–moderate	-	-	Severe, acute bronchopneumonia with foreign material	Megaesophagus	Moderate, chronic mesenteric steatitis	Aspiration pneumonia
3	Moderate	-	Moderate subacute tubular degeneration and necrosis Moderate tubular lipidosis	Marked pulmonary edema. Moderate capillary thrombosis	Moderate small intestinal crypt necrosis and regeneration	-	Hepatic lipidosis
4	Marked	-	Moderate tubular lipidosis. Mild acute tubular degeneration and proteinosis	-	Marked epithelial retention in esophagus. Marked ileitis with acanthocephalans	-	Hepatic lipidosis
5	Moderate-marked	-	Moderate-acute tubular degeneration, necrosis, and lipidosis	Severe, acute bronchopneumonia with foreign material	Pyloric stenosis	Moderate left ventricular and interventricular septal hypertrophy	Aspiration pneumonia secondary to pyloric stenosis
6	Marked	-	Moderate–severe acute tubular degeneration	Severe, acute bronchopneumonia with foreign material	Moderate necrosuppurative pancreatitis Moderate epithelial retention in stomach and esophagus	Cerebral edema with severe subacute-acute neuronal necrosis	Hepatic lipidosis
7	Moderate	-	-	Unilateral, acute bronchopneumonia Chronic histiocytic bronchointerstitial pneumonia with mineralization	Marked epithelial retention in stomach and esophagus	Necrotizing mesenteric steatitis Metastatic mineralization (lung, spleen, testes, kidney, aorta)	Bronchopneumonia
8	Marked	Severe necrotizing hepatitis Chronic lymphoplasmocytic portal hepatitis	Mild acute tubular degeneration and proteinosis	-	Marked diffuse crypt hyperplasia and few crypt abscesses Marked epithelial retention in esophagus	-	Hepatitis and hepatic lipidosis
9	Moderate	-	Moderate acute tubular degeneration	Moderate pulmonary edema Mild multifocal thrombosis	Moderate epithelial retention in stomach and esophagus	Moderate sterile pericarditis Left mammary gland abscess	Hepatic lipidosis

The cause of death or reason for euthanasia was determined by American College of Veterinary Pathologists board certified pathologists.

#### Signalment and clinical signs

The youngest animal affected was 7 months old. The ages of six of the animals were unknown as they were born in the wild and brought into human care as adults. All animals had instigating factors or comorbidities that led to clinical signs of hyporexia, weight loss, and/or constipation (Table 1). The period of final illness prior to death ranged from 2 to 75 days, with a median of 23 days. Four animals were obese at the start of illness – in all of these cases, previous attempts had been made to encourage weight loss by decreasing the diet fed by 20–25%. Inappetence was the most common initial clinical sign (7/9). All animals had anorexia and lethargy, with a median weight loss of 11% (range 0.9–20.7%) over the course of illness (Table 1). Gastrointestinal signs, including constipation (6/9), vomiting or regurgitation (6/9), and/or diarrhea (5/9), were present in most animals. Dyspnea was also common, seen in one animal at initial presentation, and developing in four others (case 2, 5–7, 9) during the course of illness. Two animals were noted to be ataxic (cases 4 and 7), and icterus was noted in one animal (case 3). Other clinical signs were more variable between individuals and were often attributable to the inciting cause of anorexia or comorbidities (Table 1).

#### Clinicopathologic findings

Baseline clinicopathologic data (representing a timepoint when each individual was healthy), as well as data from close to the start of illness, and in late illness, are reported in Tables 2, 3. The most common aberrations in biochemical analytes were increased activities in aspartate aminotransferase (AST) and alanine aminotransferase (ALT), each present in 7/9 animals (Table 2). Alterations in liver functional analytes were also seen, the most common of which included hyperbilirubinemia (5/9) and an increase in bile acids (5/9) (Table 2). Three animals had an elevation in bile acids at a baseline sample. No animals experienced a leukocytosis (total WBC > 16.7 K cells/ul) (11), although some had neutrophil left shift (5/9) and toxic change (6/9), which were attributed to inflammatory comorbidities including pneumonia (*n* = 4), mammary gland abscessation (*n* = 1), and hepatitis (*n* = 1). Anemia (PCV < 24%) (11) was noted in 3/9, and may have been secondary to chronic disease.

During the course of illness, at least one full urinalysis was performed for six pangolins, and all had glucosuria and proteinuria (Table 3). One animal (case 7) had only a urine protein measured but not a full urinalysis, and proteinuria was detected. Mild azotemia (characterized by an increase in blood urea nitrogen and/or creatinine) was noted in 6/9 animals (Table 3).

In total, triglycerides were analyzed on 17 banked serum samples and 6 plasma samples. Serum was available for all three time points for 7/9 individuals. For case 1, it was only available for baseline, and for case 7, it was only available for early and late illness. Species-specific reference intervals were not available for triglycerides, so a cut-off of 120 mg/dL was used based on extrapolation from domestic cats and cattle (13). Only one animal experienced hypertriglyceridemia during illness, and two had hypertriglyceridemia during periods where they were clinically healthy (Table 2). Grossly visible lipemia was only noted in a single sample from an animal (case 2) collected during illness, and this was subjectively graded as mild. Triglycerides were not measured on the sample where lipemia was observed in case 2, but were normal at the closest timepoint where they were measured (12 mg/dL, 8 days prior to the lipemic sample). No animals had hypercholesterolemia, but four had hypocholesterolemia (cases 4, 5, 8, 9).

For protein electrophoresis, data from all three timepoints were available for 6/9 animals. Protein electrophoresis was not performed at any point on case 1. Case 2 was missing data for late illness, and case 7 was missing baseline data. Four animals had abnormalities on their electrophoretograms. Peaks in alpha-2 globulins were seen in cases 4 and 9 at late illness, and case 2 at early illness. Hypoalbuminemia was seen in cases 4 and 9 in late illness, and in case 2 at baseline (Table 2). A mild elevation in beta globulins was seen in case 8 at early illness.

Eight of nine animals had serum measurements of vitamin E and selenium performed within 4 months of death; of these, none had low vitamin E (RI 0.26–7.2) (11), and three (cases 6, 8, 9) had low selenium only on the sample taken closest to death (range 83–156, RI 246–597) (11).

#### Imaging findings

**Figure 1:**
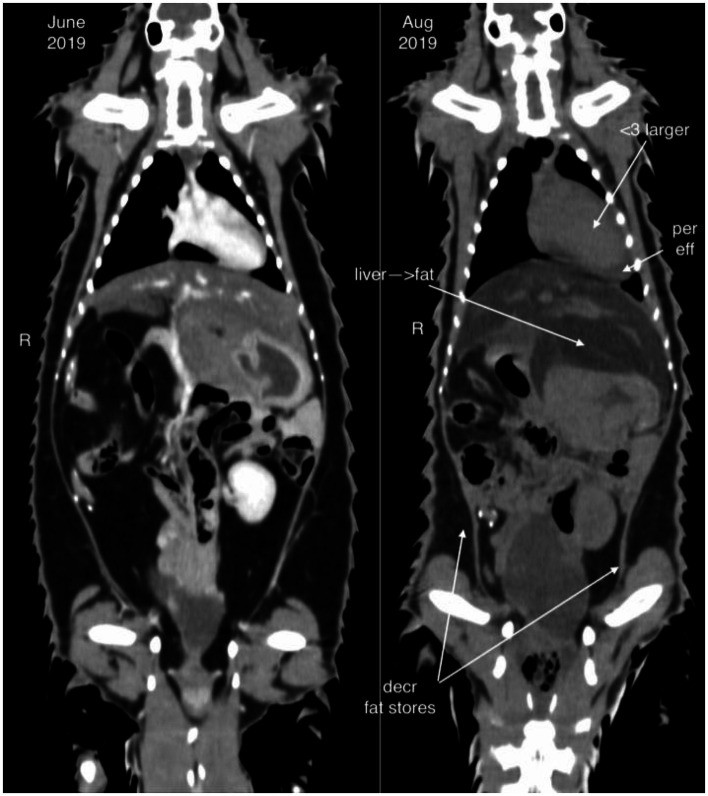
Computed tomography image of an African white-bellied pangolin (*Phataginus tricuspis*) with hepatic lipidosis (case 8) during a period of being clinically normal (image to the left, June 2019) and shortly before death due to hepatic lipidosis (image to the right, August 2019). The liver is enlarged and hypoattenuating in the period before death. Cardiomegaly, pericardial effusion, and decreased fat stores are also noted.

Diagnostic imaging included radiography (8/9), abdominal ultrasound (7/9), and computed tomography (CT, 3/9). Hepatomegaly was not appreciated on radiographs in any case, but radiographs were useful in identifying comorbidities such as pneumonia (*n* = 2) and megaesophagus (*n* = 1). On ultrasound, the liver was appreciated as being enlarged in 5/7 animals, and hyperechoic to the spleen in 2/7 animals. Computed tomography revealed the liver was severely hypoattenuating in 1/3 cases (Figure 1).

#### Comorbidities

Comorbidities included diseases that likely contributed to the onset of anorexia (Table 1), as well as disease that developed over the course of illness, the most significant of which was bronchopneumonia (*n* = 4), likely secondary to aspiration in at least three cases (Table 4).

#### Treatment

Nutritional support was administered to 7/9 animals. Gavage feeding was performed in six animals, and total parenteral nutrition in two. Two animals had feeding tubes placed surgically; one underwent endoscopic placement of a percutaneous gastrostomy tube (case 5), and the other had a gastrostomy-jejunostomy tube placed during an exploratory laparotomy (case 4). Both animals that had surgical feeding tubes died within 24 h of the placement procedure, before nutrition could be delivered. Fluid therapy with isotonic crystalloids was used in all animals. Administration was subcutaneous in eight individuals, and intravenous or intraosseous in four animals. Gastroprotectants were administered to all individuals. The most commonly used gastroprotectants were famotidine (9/9, 0.5 mg/kg PO, IO, SQ q 12 h–q 48 h, 10 mg/mL, Hikma, Berkeley Heights, NJ), and sucralfate (7/9, 250 mg–1 gram PO q 24 h, 1 gram tablets, Nostrum Laboratories Inc., Kansas City, MO). Antibiotics were also given in all cases to treat suspected or confirmed bacterial comorbidities. The most commonly administered antibiotics were ceftiofur crystalline free acid (7/9, 6–8 mg/kg SQ q 72 h, Excede for swine, 100 mg/mL, Zoetis, Parsippany, NJ), enrofloxacin (7/9, 10 mg/kg PO, SQ q 24 h, 22.7 mg/mL injectable, Baytril, Bayer, Shawness Mission, KS and 22.7 mg/mL oral, Wedgewood Pharmacy, Swedesboro NJ), and ampicillin (5/9, 20–40 mg/kg SQ, IV q 8 h–12 h, Putney, Inc., Portland, ME). Analgesic medications were administered in 8/9 cases, with buprenorphine being the most common (7/9, either 0.005–0.02 mg/kg SQ/IM q 24 h of 0.3 mg/mL injection, Par Pharmaceutical, Chesnut Ridge, NY, or 0.15–0.2 mg/kg q 24 h of Simbadol, 1.8 mg/mL, Zoetis, Parsippany, NJ). The anti-emetic maropitant was used in 7/9 cases (1 mg/kg SQ q 24 h, Cerenia, 10 mg/mL, Zoetis, Parsippany, NJ), and ondansetron was used in 5/9 (1 mg/kg PO q 12 h or 0.13 mg/kg SQ q 12 h, Aurobindo, East Windsor, NJ). Various appetite stimulants and prokinetics, including metoclopramide (0.2 mg/kg SQ, IV q 12 h, 1 mg/mL, Pharmaceutical Associates Inc., Greenville, SC), cisapride (0.5 mg/kg PO q 24 h, Wedgewood Pharmacy, Swedesboro, NJ), mirtazapine (3.75 mg PO q 72 h, Aurobindo, East Windsor, NJ), and capromorelin (1 mg/kg PO q 24 h, Entyce, 30 mg/mL, Elanco, Greenfield, IN), were used in 5/9 cases. B vitamin supplementation was administered in 7/9 (0.1mls SQ q 24 h–q 12 h, Vitamin B Complex 150, Sparhawk Laboratories, Lenexa, KS), and vitamin E supplementation was administered in 2/9 (10 units SQ, 300 IU/mL, VetOne, MWI, Boise, ID). Antioxidants such as N-acetylcysteine and s-adenosylmethionine/silybin (56.25 mg PO q 48 h, Denamarin, 225 mg tablets, Nutramax Laboratories, Lancaster, SC) were administered to three animals. Other treatments were administered based on individual clinical signs and comorbidities. Clinicians and keepers also considered the role of stressors as contributing to anorexia and attempted to reduce these as much as possible by reducing handling frequency (within the constraints of treatments) and, in one case (case 6), moving the animal to a quieter enclosure.

#### Case outcome and necropsy

**Figure 2:**
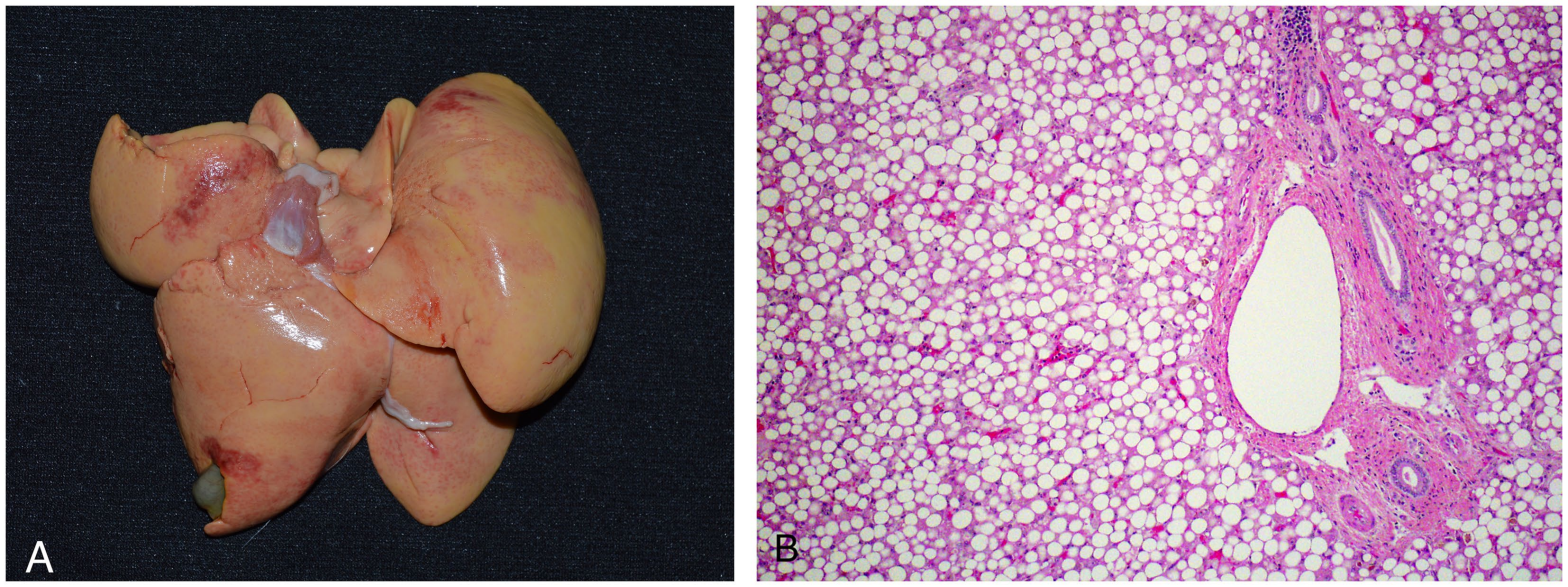
Gross **(A)** and histologic **(B)** images of an African white-bellied pangolin (*Phataginus tricuspis*) with severe hepatic lipidosis (case 3). Histologic sections are stained with hematoxylin and eosin. Grossly, the liver is enlarged, pale, and has rounded edges. Microscopically, hepatocytes are distended and distorted by large, discrete, round, colorless cytoplasmic lipid vacuoles.

**Figure 3:**
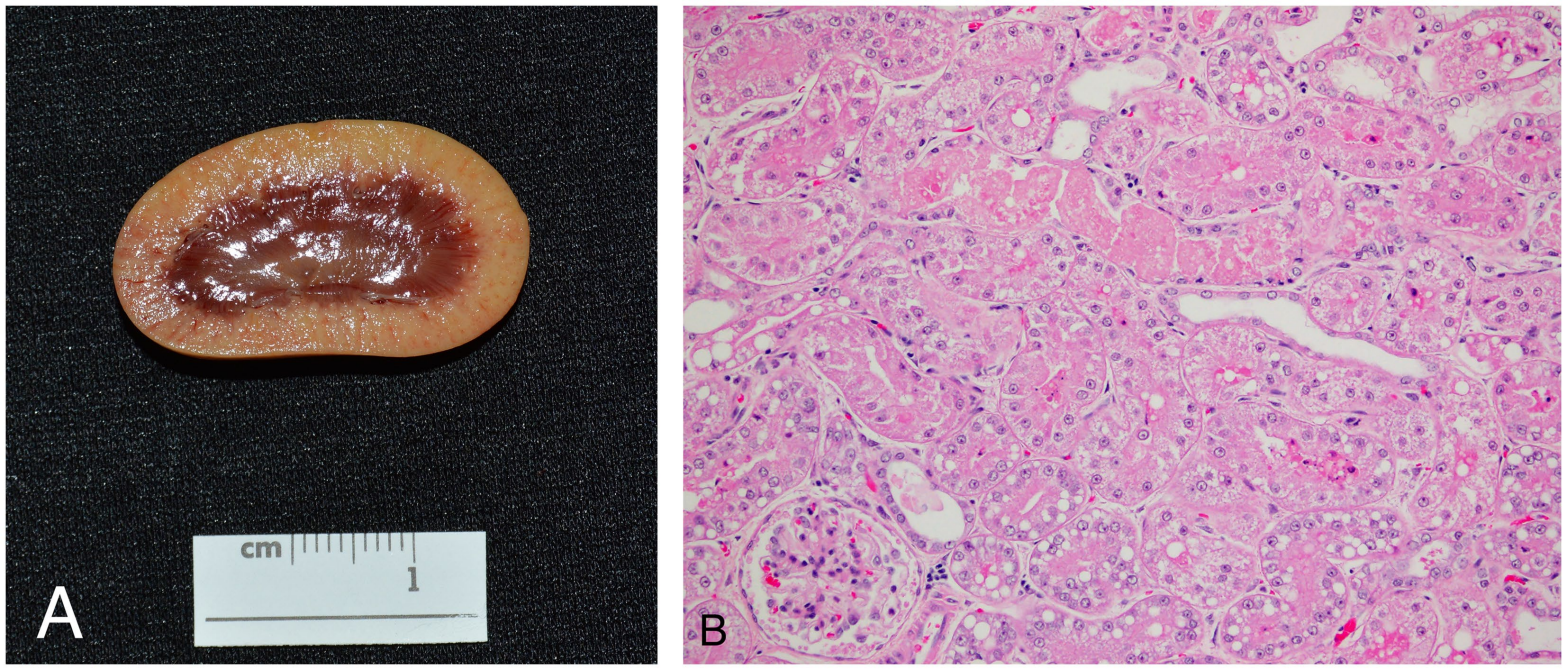
Gross **(A)** and histologic **(B)** appearance of the kidneys in an African white-bellied pangolin (*Phataginus tricuspis*) with hepatic lipidosis and nephrosis (case 3). Histologic sections are stained with hematoxylin and eosin. Grossly, the renal cortex is diffusely pale tan. Microscopically, most tubules are lined by swollen epithelial cells with pale, eosinophilic, lacey cytoplasm (hydropic degeneration), and many also have one or more, discrete, route, colorless, cytoplasmic vacuoles (lipidosis). Some tubules contain luminal hypereosinophilic, sloughed, necrotic debris, and are lined by attenuated to flattened epithelial cells. Overall findings are consistent with tubular degeneration and necrosis.

Six animals died spontaneously (cases 1–5, 7), and three animals (cases 6, 8, 9) were euthanized because of continued clinical decline despite treatment. Euthanasia was performed via an intravenous overdose of potassium chloride while animals were under inhalant anesthesia with isoflurane. On gross exam, livers were enlarged, pale, and friable (Figure 2A). Histopathologic findings are summarized in Table 4. On histologic sections stained with hematoxylin and eosin, hepatocytes were distended and distorted by large, discrete, colorless, round cytoplasmic (lipid) vacuoles (Figure 2B). In all cases, hepatocellular lipid accumulation was diffuse and uniform with no discernible zonal pattern evident on light microscopic evaluation. Seven of nine animals had renal tubular epithelial changes including lipidosis, proteinosis, degeneration and necrosis (Figure 3; Table 4). In three of these cases (cases 3, 5, 6), nephrosis was severe and likely a late contributor to clinical course. In the other 4 cases, renal lesions were mild and interpreted to be subclinical at the time of death. In five cases, the most significant histologic abnormality and primary contributor to decline/death was hepatic lipidosis. Hepatic dysfunction and resultant complications such as toxemia and organ failure led to demise. In three of the remaining cases, pneumonia was determined to be the most direct cause of euthanasia or death, including case 7 that died after 2 days of illness, that included dyspnea, with no changes in hepatobiliary analytes on bloodwork. Of the remaining two cases that were dyspneic prior to death, respiratory signs were attributed to comorbidities unrelated or subsequent to hepatic lipidosis (aspiration pneumonia (case 6) and pleural effusion and pulmonary edema (case 9), respectively). In case 8, death was multifactorial and due to the combined effects of hepatitis and severe hepatic lipidosis. Hepatitis was reflective of portal sepsis secondary to alimentary tract disease. Intestinal dysbiosis following chronic anorexia was presumed to have resulted in low grade mucosal damage that precipitated intestinal inflammation with extension to liver and portal sepsis. Other noteworthy findings included pancreatitis in 1 animal, steatitis of mesenteric adipose tissue in two cases, one of which also had metastatic mineralization, and chronic ulcerative ileitis due to acanthocephalan parasitism (family Oligacanthorhynchidae confirmed via PCR) in case 4. Frozen liver was available for vitamin E testing (Heartland Assays) for cases 1 and 4; in both these cases, concentrations were considered normal based on extrapolation from cattle (14). Giemsa and Gram stains, as well as immunohistochemistry for *Toxoplasma* sp., were performed on sections of stomach from case 1 to investigate potential underlying causes of gastric muscularis myositis. No organisms or positive immunoreactivity were seen.

## Discussion

This study describes clinical and pathologic features of hepatic lipidosis in an endangered species of pangolin while in managed care. Hepatic lipidosis was a common consequence of hypo−/anorexia in this species due to a variety of inciting/underlying etiologies. Hepatic lipidosis and consequences were reflective of generalized metabolic derangements instigated by negative energy balance, and once established proved to be refractory to treatment and an important cause of demise in these pangolins. Common clinical signs included lethargy, anorexia, diarrhea, vomiting, constipation, and dyspnea, although it was not always clear whether these clinical signs were a primary consequence of hepatic lipidosis versus other comorbidities. The most frequently administered treatments were antibiotics (as treatment for and/or prophylaxis against suspected bacterial disease), gastroprotectants, pain medications, anti-emetics, stool softeners, appetite stimulants, and fluids. In later cases from the series, increasingly aggressive therapies were attempted to reverse the negative energy balance that presumably led to the development of hepatic lipidosis. These included total parenteral nutrition delivered via a central line and placement of esophagostomy and gastrotomy tubes. Given the findings of the current study, early and aggressive nutritional therapy in ill and anorectic pangolins is recommended to prevent development of hepatic lipidosis.

This report is the first to describe hepatic lipidosis in an African species of pangolin. Hepatic lipidosis has been previously reported in a case series of necropsy findings of free-ranging Formosan pangolins and was present in 4/14 animals described, although hepatic lipidosis did not appear to be the primary cause of mortality in these animals (10). Comorbidities in Formosan pangolins with hepatic lipidosis included wound infection, pneumonia, purulent inflammation of the ventricles of the brain, and endoparasitism (10). Of these, pneumonia (*n* = 4) and endoparasitism (*n* = 1) were also present in white-bellied pangolins of the current report.

In domestic mammals, hepatic lipidosis is most well reported in cats (*Felis catus*) and cattle (*Bos taurus*) (15, 16). Other animals in which hepatic lipidosis has been described include multiple species of reptile (17, 18) and bird (19), African hedgehogs (*Atelerix albiventris*) (20), lesser hedgehog tenerecs (*Echinops telfairi*) (21), Amargosa voles (*Microtus californicus scirpensis*) (22), plains viscachas (*Lagostomus maximus*) (23), American mink (*Neogale vison*) (24), domestic ferrets (*Mustela putorius*) (25), and bottlenose dolphins (*Tursiops truncates*) (26). The main predisposing factor for hepatic lipidosis among mammals is a period of negative energy balance with an obese body condition leading to mobilization of free fatty acids from peripheral tissues into the liver and excessive accumulation of triglycerides within hepatocytes (16). This leads to oxidative damage, compression of bile duct canaliculi, and consequent hepatic dysfunction (16). About half of the pangolins in this report were obese at the start of their illness, and all had hyporexia, anorexia, and weight loss as part of their clinical course, although the inciting causes of inappetence were diverse and not always clear.

Cats are thought to be uniquely sensitive to hepatic lipidosis due to adaptation of their metabolism for a strictly carnivorous diet, including a higher requirement for amino acids, essential fatty acids, and several B-vitamins when compared to other species (27). Deficiencies in proteins and L-carnitine have been proposed to have roles in the development of hepatic lipidosis in cats (16). Common primary conditions associated with secondary lipidosis in cats include cholangitis, pancreatitis, inflammatory bowel disease, diabetes mellitus, and hyperthyroidism (16, 28). The conditions underlying anorexia in the pangolins of this report were diverse and included pneumonia, gastrointestinal disease, or pancreatitis. Potential reasons for the apparent predisposition of this species to hepatic lipidosis following anorexia need further study, but may include peculiarities of lipid and/or protein metabolism and/or nutritional deficiencies related to a non-natural diet. Two animals in this report had steatitis of mesenteric adipose tissue, one of which also had metastatic mineralization, suggestive of a potential nutritional imbalance or antioxidant deficiency, although neither of these animals had hypovitaminosis E or a low selenium. Given studies regarding the exact composition of pangolins’ natural diets are still in process, recreation in a zoological setting is currently not possible. A formulated diet exists for Formosan pangolins in managed care (5), but this is a completely separate species that has evolved in a different ecological niche from the African pangolins. Managed care for African pangolins is a challenge because sourcing palatable insect dietary items and appropriate ingredients for a formulated diet can be difficult. Historically, such constraints have contributed to poor survival rates in managed care.

Interestingly, the majority of pangolins in this report did not have hypertriglyceridemia during their period of illness. In other mammals, including cats and humans, increased blood triglycerides are a common feature of hepatic lipidosis (16, 29). This implies that the liver is still able to export triglycerides as lipoproteins, albeit to a rate insufficient to prevent buildup of triglycerides in hepatocytes. Absence of hypertriglyceridemia during hepatic lipidosis implies that the pathogenesis may involve an impairment of the liver’s ability to export triglycerides as very low density lipoproteins, leading to triglyceride accumulation in the liver. Impairment of this pathway in other species has been linked to deficiencies in amino acids and depletion of n-3 polyunsaturated fatty acids (PUFAs) (16). Further studies are required to describe and define pathogenic mechanisms of hepatic lipidosis in pangolins. The natural diet of pangolins includes insects, ants, ant pupae, and ant eggs which all have unique PUFA profiles (30). Research is needed to elucidate associations between pangolin triglyceride exportation pathways in the liver and PUFAs in their natural diet.

The most common change in hepatobiliary analytes in this case series were increased AST and ALT activities, which were seen in all but two pangolins. Cats with hepatic lipidosis generally have elevations in bilirubin, ALT, AST, and ALP, with GGT only rising later in the course of disease (31). A minority of pangolins in this case report had increased ALP or GGT, perhaps indicating that hepatocytes were damaged to a greater degree than the biliary duct epithelium. It is also possible that the location of these enzymes differs between pangolins and domestic carnivores. Of the liver functional analytes, decreased BUN, hypocholesterolemia, hyperbilirubinemia, and elevated bile acids were the most consistent changes, detected in roughly half of pangolins, indicating that animals had varying degrees of cholestasis as well as hepatocellular injury. Bile acids were elevated in several animals at baseline, and were only drastically elevated compared to baseline values in one animal. This may be due to variations in the length of time that animals were fasted before collection of samples, an indication of impaired hepatic function prior to overt disease in some animals, and/or a reflection of using an extrapolated reference interval which may not be accurate for this species. Bilirubin was a more useful marker of hepatic lipidosis, as it rose during illness compared to baseline in all animals in which it was measured. Hypoalbuminemia was relatively uncommon, although dehydration might have masked true hypoalbuminemia due to hepatic insufficiency and/or renal losses in some cases. There was one animal with a low albumin concentration at baseline; it is possible this was related to young age, as the animal was 5 months old when the baseline sample was collected. Protein electrophoresis revealed three pangolins had elevations in alpha-2 globulins during their illness, and one animal had a mild elevation in beta globulins. Acute phase proteins group within the alpha-2 globulins and beta globulins (32). Elevations in these protein fractions indicate that some animals had active inflammation, which likely stemmed from comorbidities rather than hepatic lipidosis itself, as changes were not seen in all cases, and hepatic lipidosis is not a primary inflammatory disease. Elevated AST, ALT, bilirubin, and bile acids, as well as hypocholesterolemia, in an anorectic pangolin should provide a high index of suspicion for hepatic lipidosis. In cats with hepatic lipidosis, hypoalbuminemia, hypocholesterolemia, progressive hyperbilirubinemia, and increasing serum beta-hydroxybutyrate during hospitalization are associated with a worse prognosis (33). Prognostic indicators were not identified in the current study given all cases were fatal.

Both antemortem and postmortem evidence of renal dysfunction were documented in the pangolins affected by hepatic lipidosis in this report. All pangolins with available urinalyses had glucosuria and proteinuria at one or multiple time points during their illness. Insulin resistance has been associated with hepatic lipidosis in cats, and may have been partially responsible for glucosuria in pangolins with hyperglycemia (16). However glucosuria was also seen in pangolins in the face of mild hyperglycemia or normoglycemia, supportive of potential proximal tubular dysfunction. This was further supported by histologic evidence of renal tubular degeneration and necrosis, which was severe and clinically relevant in three cases; however, not all animals with glucosuria and proteinuria had significant histologic renal lesions. Acquired Fanconi syndrome causing glucosuria and aminoaciduria has been described in humans and dogs with hepatic dysfunction secondary to copper storage disease, but to the authors’ knowledge, has not been reported in any species in conjunction with hepatic lipidosis (34, 35). Fanconi syndrome is not typically associated with histologic renal lesions, so it is not necessarily unexpected that some pangolins with proteinuria and glucosuria did not have renal lesions, or had only mild renal lesions, on necropsy (36). This potential association of Fanconi syndrome with hepatic lipidosis in pangolins warrants further investigation. When azotemia was present it was mild, suggesting these pangolins had a greater degree of tubular dysfunction than decrease in glomerular filtration rate. Histologic renal tubular changes included lipidosis, degeneration, necrosis (nephrosis) and proteinosis. Lipidosis was present in cases lacking histologic tubular damage as well as in cases with tubular degeneration and necrosis. Findings indicated that renal tubular lipidosis alone was an incidental lesion. Given the absence of glomerular abnormalities, tubular proteinosis was attributed to tubular epithelial degeneration and necrosis. In all but one case, renal lesions were acute, and findings were most consistent with renal damage being a secondary process instigated by disease in other systems. Toxemia and ischemia were postulated as likely contributors to renal tubular damage. In one case (case 3), pigmentary nephropathy associated with hyperbilirubinemia was implicated in development of nephrosis.

In the cases described here, a definitive diagnosis of hepatic lipidosis was only made on necropsy. In cats, the gold standard for antemortem diagnosis is hepatic biopsy, but this is invasive and not without risk in animals with hepatic dysfunction and thus potential coagulopathies. One animal in this report did undergo hepatic biopsy with no apparent intra-operative complications but died the day following surgery; there was no evidence of coagulopathy on necropsy. In cats with hepatic lipidosis, abdominal ultrasound generally reveals a liver hyperechoic to the falciform fat, a finding which was reported to be 91% sensitive for diagnosis of severe hepatic lipidosis (37). Liver cytology of fine needle aspirates can also be useful for diagnosis of hepatic lipidosis in mammals, although results may be unreliable (38). In the cases described herein, a diagnosis of hepatic lipidosis was suspected when pangolins presented with elevated activities of hepatocellular leakage enzymes, and ultrasound was the most reliable imaging technique at identifying features of hepatic lipidosis, including hepatomegaly and increased echogenicity.

Vitamin E and selenium both have antioxidant effects, and supplementation of vitamin E in mammals with hepatic lipidosis has been recommended by some authors (31, 39, 40). Accordingly, serum selenium and vitamin E, as well as hepatic vitamin E were evaluated in the study pangolins. No evidence of vitamin E deficiency was noted, although several animals did have decreased serum selenium. To the authors’ knowledge, few studies have examined the selenium status of domestic animals with hepatic lipidosis, although one study found no difference in plasma selenium between cows with and without hepatic lipidosis, and a review of selenium in human patients with liver disease found low selenium in association with several types of liver disease, but not fatty liver (41, 42). Low selenium was only detected in samples taken on the day of death and not earlier, suggesting decreased concentrations were temporally related to hepatic lipidosis rather than a predisposing factor. Selenium supplementation in pangolins with hepatic lipidosis may be warranted. Other antioxidants were administered to several of the animals in this report; antioxidants are frequently recommended for the treatment of hepatic lipidosis in domestic species due to the role of oxidative damage in the pathophysiology of this condition (31).

Therapy for these pangolins was extrapolated from treatment guidelines for other species. In one retrospective study of cats with hepatic lipidosis, 55% of those treated with aggressive early nutritional therapy survived, compared with 0% of those that did not, and another study reported an 86% survival rate when tube feeding was implemented early (28, 43). Both pangolins with feeding tubes placed in this report died within 24 h of placement, so unfortunately neither had time to benefit significantly from more aggressive nutritional therapy. Complications and stress of anesthesia and surgery could have detrimentally contributed to clinical course in these animals. Even though surgical placement of a feeding tube is an invasive procedure, early placement, when animals are relatively stable, may be of benefit for pangolins. Potential complications of assist feeding should also be considered. Three animals in this report had aspiration pneumonia, which is a potential complication of tube feeding. Direct contribution of tube feeding to aspiration in affected pangolins was uncertain as one of these animals had pyloric stenosis and the other had megaesophagus, conditions that also could have predisposed them to regurgitation and subsequent aspiration. In addition, various medications were used as part of the treatment protocol, and any one or combination could have impacted hepatic or renal function in the pangolins. No medications have been studied for efficacy or safety in this species, making it impossible to assess whether possible adverse drug effects could have had a role in the deaths of these animals.

Interpretations of the case findings were necessarily limited by the retrospective nature of this investigation, and small number of cases. Diagnostic approaches and treatments varied from case to case and evolved as knowledge was gained from preceding experiences. It was not always possible to definitively determine which comorbidities developed prior to or following the onset of hepatic lipidosis, and which clinical signs were primarily attributable to hepatic lipidosis versus other illnesses. It was impossible to objectively assess and compare the utility or success of particular diagnostic modalities or treatments. Animal care was prioritized, and decisions were made based on best practices and veterinarians’ discretion at the time. With experience, animal care staff became more vigilant in monitoring. Consequently, later cases were treated more aggressively than earlier cases. An additional constraint of the current report and common obstacle of investigations involving non-domestic, zoo animals was the lack of published clinicopathologic reference intervals for this species. Other published studies on pangolin blood analyte data are limited due to their descriptive presentation, or because they are from wild pangolins of a different species (44, 45). For the current report, the decision was made to instead use the medical database ZIMS. However, reference intervals generated by this system may suffer from inaccuracies due to small sample size and inclusion of potentially ill animals.

This report describes a syndrome of metabolic derangements instigated by negative energy balance and manifested as hepatic lipidosis in a managed care population of white-bellied pangolins. The syndrome may be initiated by a variety of underlying diseases processes or stressors and carries a poor prognosis. Suggestive antemortem findings indicative of hepatic lipidosis include elevated hepatobiliary leakage enzymes, biochemical evidence of decreased liver function, an enlarged, hyperechoic liver on ultrasound, and, uniquely, glucosuria and proteinuria. Lipidomic studies of this species would be valuable in determining inherent metabolic factors that may predispose pangolins to hepatic lipidosis. In addition, more clinical investigation is warranted to improve antemortem diagnosis, determine effective treatments, and investigate prognostic indicators. Based on extrapolation from domestic species, early, aggressive nutritional support is warranted when veterinarians are presented with an anorectic pangolin.

## Data Availability

The original contributions presented in the study are included in the article/supplementary material, further inquiries can be directed to the corresponding author.
